# Targeting HER2 genomic alterations in non-small cell lung cancer

**DOI:** 10.1016/j.jncc.2021.04.001

**Published:** 2021-05-03

**Authors:** Jie Zeng, Weijie Ma, Richard Benjamin Young, Tianhong Li

**Affiliations:** a Division of Hematology/Oncology, Department of Internal Medicine, University of California Davis School of Medicine, University of California Davis Comprehensive Cancer Center, Sacramento, CA, USA; b Department of Respiratory Medicine, Shanghai Tenth People's Hospital, Tongji University School of Medicine, Shanghai, China

**Keywords:** HER2, ERBB2, Genomic alterations, Biomarkers, Non-small cell lung cancer

## Abstract

Oncogenic mutations and amplifications in the erythroblastic oncogene B (*ERBB2*), or human epidermal growth factor receptor 2 (*HER2*), have emerged as distinct oncogenic drivers and drug targets in non-small cell lung cancer (NSCLC). Each genomic alteration occurs in 2–4% of NSCLC by next generation sequencing and is associated with constitutive HER2 activation. The most common *HER2* mutations in NSCLC are exon 20 mutation A775_G776insYVMA mutation in the kinase domain and S310F mutation in the extracellular domain. Unlike in breast and gastric cancer, HER2 protein overexpression in NSCLC is not validated to be a biomarker predictive of clinical response to HER2-targeted agents. High HER2 protein overexpression by immunohistochemistry (3^+^) only occurs in 2–4% of NSCLC. Until now HER2-targeted agents (such as afatinib and ado-trastuzumab emtansine) only demonstrate modest clinical activity in patients with *HER2*-mutant NSCLC. Retrospective studies show concern for inferior clinical benefit of immune checkpoint inhibitors in *HER2*-mutated NSCLC. Therefore, platinum-based chemotherapy with or without an anti-angiogenesis inhibitor remains the first line standard treatment for this patient population. In May 2020 trastuzumab deruxtecan (T-DXd) received the U.S. Food and Drug Administration breakthrough therapy designation for *HER2*-mutant metastatic NSCLC, and was added as an option for *HER2*-mutant NSCLC to the NCCN guidelines V1.2021. A global phase III study of pyrotinib compared to docetaxel as a second line therapy for advanced NSCLC harboring *HER2* exon 20 mutations was just opened for enrollment in September 2020. In this review, we highlight the current knowledge and perspectives on targeting-*HER2* genomic alterations in NSCLC.

## 1 Introduction

Lung cancer is the leading cause of cancer related death for both men and women worldwide, with nearly 1.8 million deaths each year^1^. Non-small cell lung cancer (NSCLC) accounts for approximately 80–85% of all lung cancer cases. NSCLC is among the most genomically diverse and deranged of all cancers, which creates tremendous challenges for both prevention and treatment strategies. Increasingly over time, driver oncogenic mutations have been described by clinical use of multiplex next generation sequencing (NGS). Currently, both the National Comprehensive Cancer Network (NCCN) and American Society of Clinical Oncology (ASCO) guidelines recommend testing for mutations in *EGFR, BRAF, MET, NTRK* and gene fusions (or rearrangements) in *ALK, ROS1, RET* and *NTRK* for all NSCLC tumors that contain an adenocarcinoma component, regardless of histologic grade, dominant histologic subtype, clinical characteristics or demographic information^2^^,^^3^. Genomic alterations, mainly amplification and mutations, in the erythroblastic oncogene B (*ERBB2*) or human epidermal growth factor receptor 2 (*HER2*) have emerged as distinct oncogenic drivers in 2–4% of NSCLC^4^, ^5^, ^6^, ^7^, ^8^. Furthermore, HER2 protein overexpression is detected in less than 20% of patients with NSCLC and is associated with poor prognosis in both resected and advanced NSCLC^9^. Unlike in breast and gastric cancer, in NSCLC HER2 protein overexpression is not validated to be a biomarker predictive of clinical response to HER2-targeted agents. In this review, we summarize the current knowledge and perspectives on targeting *HER2* genomic alterations in NSCLC.

## 2 *HER2* gene and its protein product

**Fig. 1:**
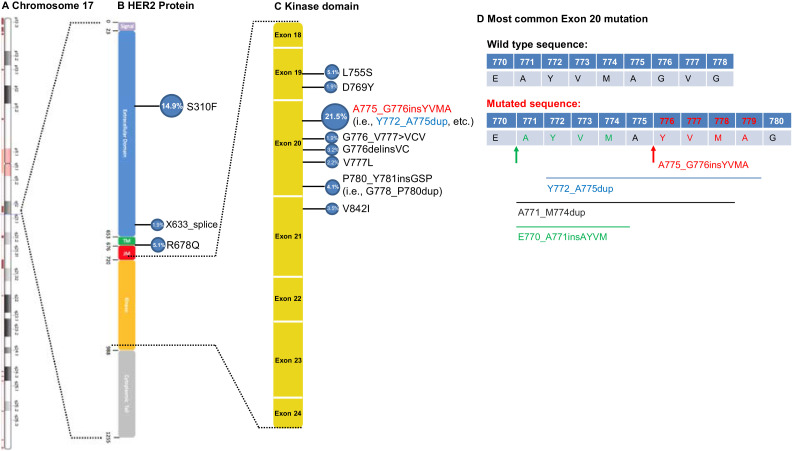
*HER2* mutations in NSCLC. (A) Schema illustrates the location of *HER2* (*ERRB2*) gene in chromosome17. (B) and (C) Distribution and frequency of reported *HER2* mutations collected from Foundation Medicine report and the cBioPortal database in the entire gene (B) and tyrosine kinase domain (C). (D) Depending on the proposed molecular mechanisms, the most common HER2 mutation A775_G776insYVMA (an insertion of the YVMA between A775 and G776) is also named Y772_A775dup (a duplication of Y772-A775 YVMA), E770_A771insAYVM (an insertion of the AYVM between E770 and A771), or A771_M774dup (a duplication of A771_M774 AYVM) in the scientific literature .

*HER2*, also known as proto-oncogene *Neu, ERBB2* (human), or cluster of differentiation 340 (CD340), is located at 17q12 of the human chromosome and is 28,515 base pairs in length (Fig. 1A). It was first identified in an ethylnitrosourea (ENU) induced rodent glioblastoma cell line and is highly homologous to the *HER1* gene, which encodes the human epidermal growth factor receptor (EGFR). The *HER2* transcript is a 4626-nucleotide mRNA that encodes the plasma membrane-bound receptor tyrosine kinase HER2 protein. The HER2 protein is 185 kilodaltons (kDa) and composed of 1255 amino acids. Similar to other HER family members, the HER2 protein has three major domains; the extracellular ligand binding domain (the first 652 amino acids), the transmembrane domain (amino acids 653-675) and the intracellular domain (amino acids 676-1255) (Fig. 1B).

**Fig. 2:**
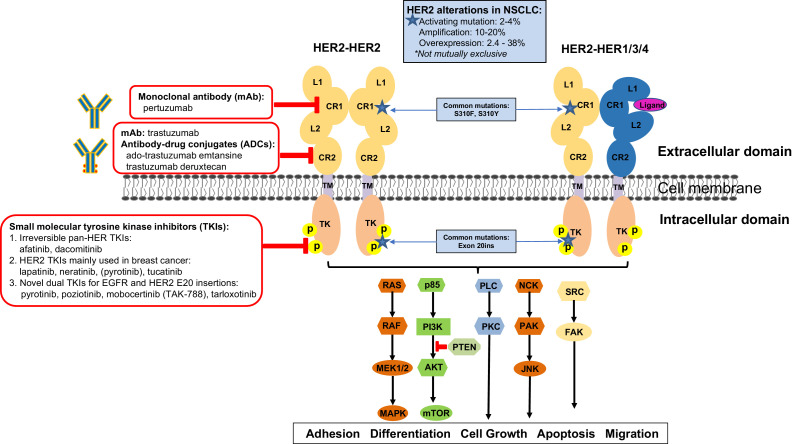
HER2 targets and HER2-targeted drugs. HER2 alterations in NSCLC included activating mutation (2–4%), amplification (10–20%) and protein overexpression (2.4 - 38%). HER2 can form either homodimers or heterodimers with another HER family member (EGFR, HER3 or HER4) upon ligand binding. This triggers the autophosphorylation of tyrosine residues within the cytoplasmic domain of receptors and initiates a variety of signaling pathways that play important roles in various cellular functions including adhesion, differentiation, growth, apoptosis and migration. Monoclonal antibodies and antibody-drug conjugates, such as trastuzumab, pertuzumab, ado-trastuzumab emtansine, and trastuzumab deruxtecan, bind to the extracellular domain of HER2. Small molecular TKIs, such as afatinib, dacomitinib, lapatinib, neratinib, pyrotinib, poziotinib, tucatinib, mobocertinib, and tarloxotinib, bind to the intracellular domain of HER2. Abbreviations: CR, cysteine-rich regions; L, ligand binding regions; TM, transmembrane; TK, tyrosine kinase.

HER2 is normally expressed in the fetal period and at very low levels within some tissues in adults. HER2 plays a critical role in NSCLC development and progression by forming heterodimers with other HER family members (EGFR or HER1, HER2 and HER4). Additionally, HER2 may form homodimers when it is highly expressed^10^. HER2 can increase the activated HER proteins’ affinity to ligands, affect the stability of the receptor and induce the amplification of the HER2 signal cascade. While the members of the HER family contain similar domains, each of the four proteins (EGFR, HER2, HER3 and HER4) have distinct properties. For example, HER2 has strong kinase activity but has no identified ligand; while HER3 is devoid of kinase activity due to substitutions in crucial tyrosine kinase (TK) domain residues^11^. The HER family members can form either homodimers or heterodimers upon ligand binding (Fig. 2). This triggers the autophosphorylation of tyrosine residues within the cytoplasmic domain of receptors and initiates a variety of signaling pathways. These receptors work through a complex array of secondary messengers and play important roles in various cellular functions including adhesion, differentiation, growth, apoptosis and migration^12^. Overexpression of HER2 alone, or in co-overexpression with EGFR, could enhance down regulation of EGFR tyrosine kinase activity and induce aggressive tumor growth^13^.

## 3 Testing for HER2 alterations in NSCLC

HER2 alterations, which include mutations, gene amplification, and protein overexpression, have been detected in over 100 cancer types and are associated with poor prognosis^14^, ^15^, ^16^. There are significant variations in the type and frequency of HER2 alterations in different cancer types. These variations may be attributed to different tumor biology, tumor heterogeneity, sample size, race and ethnicity, detection methods, and definitions of overexpression. Table 1 summarizes the reported HER2 alterations in human NSCLC specimens as compared to other major cancer types including breast, gastric, gastroesophageal junction (GEJ) adenocarcinomas, and colon cancer.

**Table 1:** The prevalence of HER2 alterations in solid tumors.

**Tumor Type**	***HER2* mutations (by PCR or NGS)**	***HER2* gene amplification (by FISH or NGS)**	**HER2 protein 2^+^ or 3^+^ (by IHC)**
Lung	2–4%^7^^,^^22^	2–4% by NGS and 10–20% by FISH^23^^,^^99^	2–38%^23^^,^^99^^,^^100^, and 3^+^ in 2–4%^30^^,^^99^^,^^101^
Breast	3–4%^102^^,^^103^	18%-47%^104^^,^^105^	15%-35%^104^^,^^106^
Gastric	0–2%^18^^,^^107^	15–20%^18^^,^^107^	10%-46%^18^^,^^107^
Colon	5–7%^108^, ^109^, ^110^	5–7%^109^, ^110^, ^111^	14%^110^

Abbreviations: FISH, fluorescent in situ hybridization; IHC, immunohistochemistry; NGS, next-generation sequencing; PCR, polymerase chain reaction.

Currently, there is no guideline established for HER2 testing in lung cancer. This contrasts with breast, gastric and GEJ adenocarcinomas, which have amplification of wild‐type HER2 with protein overexpression in up to 20% of cases. *HER2* amplification, defined as a HER2/CEP17 ratio ≥2, was present in 10–20% of cases by fluorescence in situ hybridization (FISH), but only occurs in 2–4% of NSCLC cases by NGS^14^^,^^15^^,^^17^. Thus, the HER2 testing guidelines for gene amplification and protein overexpression in breast cancer, gastric cancer, and GEJ adenocarcinomas^18^ are unlikely to be helpful for NSCLC. *HER2* amplification is shown to increase the invasiveness of NSCLC cells *in vitro* and can elicit the constitutive activation of both HER2 and ligand-independent EGFR^19^. Higher *HER2* amplification numbers are associated with increased clinical response to HER2-targeted agents in case reports and early phases of clinical trials when studied in different cancer types^20^^,^^21^. The optimal cutoff for predicting clinical benefit of *HER2* amplification in NSCLC remains to be defined through clinical trials.

In addition to gene amplification, *HER2* mutations were identified in 2–4% of NSCLC tumors by routine, clinical use of comprehensive molecular profiling via NGS^7^^,^^22^^,^^23^. Like *EGFR* mutations, the frequency of *HER2* mutations is significantly increased in non-smokers, women, persons of Asian descent, and adenocarcinoma NSCLCs^8^. *HER2* mutations are generally mutually exclusive with other oncogenes in NSCLC. The incidence of *HER2* mutations can be as high as 6.7% in *EGFR/ALK/ROS1*-negative NSCLC^24^, especially in those who were negative for *EGFR*. Pan-cancer analysis shows that the most common *HER2* mutations are S310F/Y (11.0%), A775_G776insYVMA (5.7%), L755P/S (4.6%), V842I (4.4%), and V777L/M (4.0%). Although notably, the frequency and hotspots of *HER2* mutation vary among different cancer types^15^. In breast cancer and colorectal cancer, the most common *HER2* mutations are in exon 19 and exon 21, respectively. The most common *HER2* mutations in lung cancer occur in exon 20. Among the exon 20 mutations, the most common mutation is *ERBB2 (HER2)* A775_G776insYVMA. These *HER2* mutations lead to constitutive activation of the receptor and the downstream activation of the phosphatidylinositol 3‑kinase (PI3K)/protein kinase B (AKT) and Ras/mitogen activated protein kinase (MAPK) signaling pathways^7^^,^^24^. *HER2* exon 20 insertion mutations and exon 19 L755P mutation are resistant to most HER2 TK inhibitors (TKIs) due to an acquired sterically hindered drug binding pocket^25^. Mutations in the extracellular domain of the *HER2* gene are oncogenic and associated with sensitivity to treatment with HER2 inhibitors^26^. There are many genomic alterations in the *HER2* gene identified by NGS in NSCLC. Due to the rare frequency, most of the studies contained small sample sizes. Table 2 summarizes the types and frequencies of *HER2* mutations in NSCLC reported from the two largest published or publicly available sequencing databases (Foundation Medicine, which contains both primary and metastatic tumors^14^, and cBioPortal [The Cancer Genome Atlas] dataset, which contains mainly primary tumors^27^. Furthermore, we summarized the known responses of each *HER2* mutation to HER2-targeted therapy into a quick clinical reference for practicing physicians. The most common *HER2* mutations in lung cancer occur in exon 20 (38.3%), followed by S310F mutations (14.9%) in the extracellular domain. The top “hotspot” *HER2* mutations with frequencies are illustrated in Fig. 1C. The molecular mechanisms that lead to these “hotspot” *HER2* mutations are unknown. *ERBB2* (*HER2*) A775_G776insYVMA is also named Y772_A775dup, E770_A771insAYVM, or A771_M774dup, in the scientific literature depending on the hypothetical explanation for the molecular mechanism of the YVMA insertion (illustrated in Fig. 1D). A recent large retrospective study shows that not only the *HER2* mutation variants, but also co-mutations, affect the response to afatinib in *HER2*-mutant NSCLC. TP53 is the most common co-mutation in HER2-mutant NSCLC. Co-mutations in TP53 and the PI3K/AKT/mTOR pathway confer additional resistance to anti-HER2 treatments in NSCLC. The underlying mechanisms and implication for treatment remain unknown^28^. Liquid biopsy for tumor genomonic profiling of plasma cell free DNA is being used more frequently to detect *HER2* genomic alterations in many solid cancer types, including NSCLC^29^.

**Table 2:** Reported *HER2* mutations in NSCLC. A total of 316 case were collected from Foundation Medicine report (N=165) and cBioPortal (N=151). Blue highlights the oncogenic mutations occurred in at least 6 (1.9%) cases and are illustrated in Fig. 1C.

***ERBB2* Mutation**	**Exon**	**Foundation Medicine****^14^**	**cBioPortal****^27^**	**Common Mutations (%)**	**Oncogenic function**	**Drug Response**	**Outcome (PFS)****^16^**
M45V	2	0	1	1	NA	NA	NA
R47H	2	0	1	1	Benign	NA	NA
V94I	3	0	1	1	Benign	NA	NA
P122L	3	0	4	4	Benign	NA	NA
G152V	4	0	1	1	Oncogenic	NA	NA
X192_splice	5	0	1	1	NA	NA	NA
K200N	5	0	2	2	Benign	NA	NA
X215_splice	6	0	2	2	Oncogenic	NA	NA
G222C	6	0	2	2	NA	NA	NA
X254_splice	7	0	1	1	NA	NA	NA
D277Y	7	0	1	1	Oncogenic	NA	NA
G292C	7	0	1	1	Oncogenic	NA	NA
G292C	7	0	1	1	Oncogenic	NA	NA
X300_splice	8	0	4	4	NA	NA	NA
N302K	8	0	1	1	Oncogenic	NA	NA
V308M	8	0	1	1	Oncogenic	NA	NA
G309A	8	1	0	1	Oncogenic	NA	NA
S310F	8	39	8	47 (14.9%)	Oncogenic	Sensitive	Ado-trastuzumab emtansine (5 mo)^71^
S310Y	8	5	0	5	Oncogenic	Sensitive	Afatinib (5 or 8 mo)^112^^,^^113^
N319D	8	1	0	1	NA	NA	NA
Q329L	8	0	2	2	NA	NA	NA
S335C	8	0	2	2	Oncogenic	NA	NA
R340P	9	0	1	1	Benign	NA	NA
R351L	9	0	3	3	NA	NA	NA
Q396K	10	0	1	1	Benign	NA	NA
X408_splice	11	0	2	2	NA	NA	NA
S418T	11	0	2	2	Benign	NA	NA
G537S	13	0	1	1	NA	NA	NA
G549W	13	0	2	2	NA	NA	NA
X633_splice	15	$2	6	6 (1.9%)	NA	NA	NA
S649T	15	0	1	1	NA	NA	NA
L651V	17	0	1	1	Oncogenic	NA	NA
V659D	17	0	1	1	NA	NA	NA
V659E	17	0	4	4	Oncogenic	Sensitive	Lapatinib (5 mo)^114^
G660D	17	0	0	0	Oncogenic	Sensitive	Afatinib (16 mo)^115^
I661V	17	0	1	1	Benign	NA	NA
R678Q	17	16	$2	16 (5.1%)	Oncogenic	NA	NA
Q680H	17	0	1	1	Benign	NA	NA
V697L	18	0	1	1	Oncogenic	NA	NA
Q711H	18	0	1	1	Benign	NA	NA
G727A	18	0	1	1	Oncogenic	NA	NA
T733I	18	1	0	1	Oncogenic	NA	NA
L755A	19	0	1	1	Oncogenic	Sensitive	Neratinib (3.6 mo)^32^
L755P	19	0	1	1	Oncogenic	Sensitive	Neratinib (14.8 mo); Poziotinib (7 mo)^15^^,^^32^
L755S	19	15	1	16 (5.1%)	Oncogenic	Sensitive	Neratinib (12.7 mo)^32^
E766Q	19	0	1	1	NA	NA	NA
I767M	19	1	0	1	NA	NA	NA
D769H	19	1	0	1	Oncogenic	NA	NA
D769Y	19	6	$2	6 (1.9%)	Oncogenic	Sensitive	Afatinib (PR 7mo)^46^
Y772_V773insLMAY	20	1	0	1	NA	NA	NA
A775_G776insSVMA	20	1	0	1	Oncogenic	NA	NA
A775_G776insV	20	1	0	1	NA	NA	NA
A775_G776insYVMA (i.e., Y772_A775dup, M774_A775insAYVM, E770delinsEAYVM)	20	31	37	68 (21.5%)	Oncogenic	Sensitive	Ado-trastuzumab emtansine (12 mo); Lapatinib, trastuzumab, and bevacizumab (7 mo); Afatinib (PR 3 mo-7 mo; SD 10 mo)^28^^,^^46^^,^^116^^,^^117^; pyrotinib (CR, PR, SD up to 23.4 mo)^118^
G776_V777>AVCV	20	1	0	1	Oncogenic	NA	NA
G776_V777>AVGCV	20	1	0	1	Oncogenic	NA	NA
G776_V777>AVGSGV	20	1	0	1	NA	NA	NA
G776_V777>VCV	20	6	$2	6 (1.9%)	Oncogenic	NA	NA
G776_V777insVC	20	0	0	0	Oncogenic	Sensitive	Ado-trastuzumab emtansine (8.5 mo)^72^
G776C	20	2	0	2	Oncogenic	Sensitive	Pyrotinib (PR)^118^
G776delinsAVGC	20	0	1	1	NA	NA	NA
G776delinsLCT	20	0	0	0	Oncogenic	Sensitive	Afatinib (PR 7 mo)^119^
G776delinsVC	20	1	10	11 (3.2%)	Oncogenic	Sensitive	Afatinib (PR 12 mo)^120^
G776L	20	0	0	0	Oncogenic	Sensitive	Afatinib (4 mo); afatinib and paclitaxel (9 mo)^121^
G776V	20	4	0	4	NA	NA	NA
V777_G778insC	20	1	0	1	Oncogenic	NA	NA
V777L	20	7	$2	7 (2.2%)	Oncogenic	Sensitive	Osimertinib and afatinib (4 wks)^122^
V777M	20	0	1	1	Oncogenic	NA	NA
G778S	20	1	0	1	NA	NA	NA
G778_Y779insGSP	20	0	0	0	Oncogenic	Sensitive	Pyrotinib (∼5.5 mo)^123^
P780_Y781insGSP (i.e., G778_P780dup)	20	7	6	13 (4.1%)	Oncogenic	Sensitive	Afatinib (25 mo)^46^ (3 mo)^99^ (4 mo)^124^
L786V	20	0	0	0	Oncogenic	Sensitive	Neratinib (3.7 mo)^32^
N813D	20	0	0	0	Oncogenic	Sensitive	Afatinib (SD 4 mo)^125^
R840W	21	0	1	1	Oncogenic	NA	NA
V842I	21	11	$2	11 (3.5%)	Oncogenic	NA	NA
T862A	21	2	0	2	Oncogenic	NA	NA
R896G	22	0	0	0	Oncogenic	Sensitive	Afatinib (14 mo)^126^
W906*	23	0	1	1	NA	NA	NA
Q943*	23	0	2	2	NA	NA	NA
G1015E	25	0	1	1	NA	NA	NA
E1021Q	25	0	4	4	Oncogenic	NA	NA
X1054_splice	25	0	3	3	NA	NA	NA
G1057V	25	0	3	3	Benign	NA	NA
S1134N	26	0	1	1	NA	NA	NA
**Total Case No.**		**165**	**151**	**316**			

Abbreviations: CR, complete response; mo, months; NA, not available; PFS, progression-free survival; PR, partial response; SD, stable disease.

Of note, *HER2* mutations are rarely associated with *HER2* amplification^30^. *HER2* mutation or gene amplification usually does not demonstrate HER2 protein overexpression^30^. Each of these three *HER2* genomic alterations is associated with clinical response to HER2-targeted therapy^18^^,^^21^. Thus, these *HER2* genomic alterations define distinct tumor subtypes, and they are being evaluated as independent biomarkers for HER2-targeted therapy.

## 4 HER2-targeted therapies

**Fig. 3:**
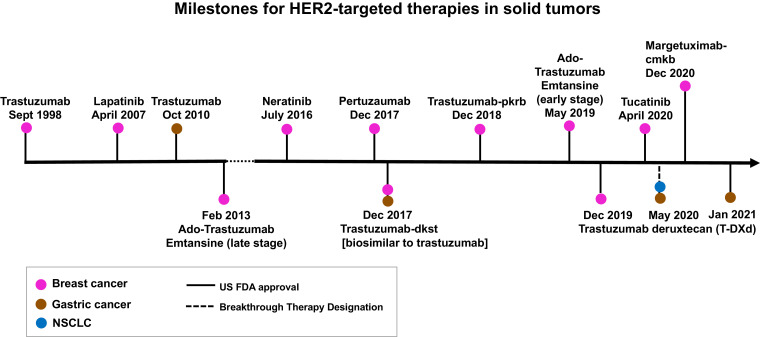
Milestones for HER2-targeted therapy in solid tumors. Currently available HER2-targeted drugs were approved for breast cancer (pink) and gastric (brown) cancer. Trastuzumab deruxtecan (T-DXd, formerly DS-8201a) is the first HER2-targeted drug which received the U.S. FDA therapy designation for *HER2*-mutant metastatic NSCLC (blue) in May 2020. On December 16, 2020, margetuximab-cmkb in combination with chemotherapy^97^^,^^98^ joins trastuzumab deruxtecan, combination of tucatinib, capecitabine and trastuzumab treatment, and combination of neratinib, capecitabine and trastuzumab treatment as third-line treatment options for adult patients with HER2-positive metastatic breast cancer.

All HER2-targeted agents (including antibodies, small molecular tyrosine kinase inhibitors and antibody drug conjugates) developed to date (Fig. 2) were developed for amplification and protein overexpression of the *HER2* wild-type genes in breast and gastric/ GEJ adenocarcinoma. Fig. 3 summarizes the milestones of the U.S. Food and Drug Administration (FDA) approval of these agents. For the rare subset of *HER2*-mutant breast cancer, neratinib demonstrates a clinical benefit rate of 31–40% in two phase II clinical trials (MutHER and SUMMIT)^31^^,^^32^. Although ado-trastuzumab emtansine is recommended as a treatment option for *HER*2-mutant NSCLC by NCCN guidelines (category 2B)^3^, currently available HER2-targeted agents only demonstrate modest clinical activity in patients with HER2-positive NSCLC after platinum-containing chemotherapy (Table 3). This is in sharp contrast to the efficacy of first line targeted therapy for NSCLC harboring other driver mutations, such as *EGFR, ALK, ROS1* and *BRAF*, and *NTRK*^33^^,^^34^. Table 4 summarizes the FDA-approved HER2-targeted drugs for breast cancer. Further studies are needed to understand the molecular and cellular mechanisms driving HER2-positive NSCLC and to develop treatment strategies for these patients. The following section reviews current knowledge and ongoing studies for targeting HER2-positive NSCLC.

**Table 3:** Reported clinical activity of HER2-targeted therapy in phase II studies in patients with NSCLC.

**Drug class**	**Drug name**	**Name (Clinical trial identifier)**	**Phase**	**No. of patients**	**Study subject**	**Biomarker(s)**	**Clinical Efficacy**			**Author (year) (reference)**
							**ORR (%)**	**PFS (mo)**	**OS (mo)**	
TKI monotherapy	Afatinib	NICHE (NCT02369484)	II	13	Advanced NSCLC	*HER2* exon 20 mutations	7.7%	4	NA	Dziadziuszko R (2019)^42^
	Dacomitinib	A7471017 (NCT00818441)	II	30	Advanced NSCLC	*HER2* mutation or amplification	11.5%	3	9	Kris MG (2015)^17^
	Neratinib	SUMMIT (NCT01953926)	II	26	Advanced solid tumors	*HER2* or *EGFR* exon 18 mutations	3.8%	5.5	NA	Hyman DM (2018)^32^
	Poziotinib	ZENITH20 (NCT03318939)	II	90	Recurrent or metastatic NSCLC	*HER2* exon 20 Insertion mutations (cohort 2)	27.8% (DCR of 70%)	5.5	NA	Ternyila D (2020)^37^
	Pyrotinib	HR-BLTN-Ⅱ-NSCLC (NCT02834936)	II	60	Recurrent or metastatic NSCLC	*HER2* mutations	30.0%	6.9	14.4	Zhou C (2020)^127^
	Pyrotinib	SHRUS 1001 (NCT02500199)	I/II	31	Advanced solid tumors whose disease progressed on prior HER2 targeted therapy	*HER2* gene amplification (FISH), overexpression (IHC 3^+^) or mutation	13.0%	5.3	NA	Li BT (2020)^123^
	Tarloxotinib	RAIN-701 trial (NCT03805841)	II	9	*HER2* mutant NSCLC	*HER2* mutation	PR: 22%	SD: 44%	NA	Liu S (2021)^66^
TKI combination	Neratinib + Temsirolimus	PUMA-NER-4201 (NCT01827267)	II	43	*HER2* mutant NSCLC	*HER2* mutations	14%	4	15.1	Li BT (2021)^128^
	Neratinib + Trastuzumab	PUMA-NER-5201 (NCT01953926)	II	52	Solid tumors	*HER2* or *EGFR* exon 18 mutation	8%	4.1	NA	
mAb	transtuzumab	NA	II	51	Advanced or metastatic NSCLC	*HER2* gene amplification (FISH) or overexpression (IHC 2/3^+^)	36.0%	6.3	NA	Gatzemeier U (2004)^68^
mAb combination	Trastuzumab + Patozumab	MyPathway (NCT02091141)	II	16	Advanced NSCLC	*HER2* mutation	13.0%	NA	NA	Hainsworth J (2018)^129^
mAb combination with chemotherapy	Trastuzumab + Carboplatin and Paclitaxel	ECOG 2598	II	139	Advanced NSCLC	HER 2 overexpression (IHC 1^+^-3^+^)	24.5%	3.25	NA	Langer CJ (2004)^67^
	Trastuzumab + Cisplatin and Gemcitabine	NA	II	103	Untreated Advanced NSCLC	HER2 overexpression (IHC 1^+^-3^+^)	36%	6.1	NA	Gatzemeier U (2004)^68^
	Trastuzumab + Docetaxel	NA	II	13	Advanced NSCLC	HER2 overexpression (IHC 2^+^, 3^+^)	NA	5.7	NA	Lara PN (2004)^69^
ADC	Ado-Trastuzumab Emtansine	NCT02289833	II	29	Advanced or metastatic NSCLC	HER2 IHC 2^+^	0%	2.6	12.2	Stinchcombe T (2017)^130^
				20		HER2 IHC 3^+^	20%	2.7	12.1	
	Ado-Trastuzumab Emtansine	MSK #15-335 (NCT02675829)	II	49	Advanced or metastatic lung adenocarcinomas	*HER2* gene amplification (NGS/FISH) or mutations	51%	5	NA	Li BT (2020)^131^
	Trastuzumab deruxtecan (DS-8201)	DS8201-A-U204 (NCT03505710)	II	42	Metastatic NSCLC	HER2 over-expression or mutation	61.9% (CR: 2.4%)	14.0	NA	Smit EF (2020)^78^

Abbreviations: ADC, antibody-drug conjugate; bid, twice daily; CR, complete response; DCR, disease control rate; DFS, disease-free survival; FISH, fluorescence in situ hybridization; IHC, immunohistochemistry; mAb, monoclonal antibody; mo, months; NA, not available; NSCLC, non-small cell lung cancer; NGS, next-generation sequencing; PR, partial response; qd, once daily; q3w, every 3 weeks; SD, stable disease; TKI, tyrosine kinase inhibitor; wk, week.

**Table 4:** Summary of FDA-approved HER2-targeted drugs for breast cancer.

**Drug class**	**Drug name**	**Name (Clinical trial identifier)**	**Phase**	**No. of patients**	**Study subject (women only)**	**Regimen**	**Clinical Efficacy**			**FDA approval date**	**Author (Year) (reference)**
							**ORR (%)**	**PFS (mo/wk)**	**OS (mo/wk)**		
**TKIs**	Lapatinib	EGF100151 (NCT00078572)	III	399	HER2 over-expressed advanced or metastatic breast cancer patients	lapatinib (1250 mg/day) + capecitabine (2000 mg/m^2^/day) on days 1–14 of a 21-day cycle	23.7%	27.1 wk	75 wk	3/13/2007	Cameron D (2008, 2010)^132^^,^^133^
						capecitabine (2500 mg/m^2^/day) on days 1–14 of a 21-day cycle	13.9%	4.4 mo	64.7 wk		
	Pyrotinib	NCT02422199	II	128	Chinese patients with HER2-positive relapsed or metastatic breast cancer previously treated with taxanes, anthracyclines, and/or trastuzumab	pyrotinib (400 mg) + capecitabine (1000 mg/m^2^) orally	78.5%	18.1 mo	NA	8/12/2018	Ma F (2019)^52^
						lapatinib 1250 mg + capecitabine 1000 mg/m^2^ orally	57.1%	7.0 mo	NA		
	Neratinib	NALA (NCT01808573)	III	621	1. Metastasis breast cancer, stage IV. 2. HER2 overexpression or gene-amplified tumor IHC 3^+^ or 2^+^. 3. Prior treatment with at least two HER2-directed regimens	Arm A: neratinib (240 mg qd) + capecitabine (1500 mg/m^2^ daily, 750 mg/m^2^ bid)	32.8%	5.6 mo	29 mo	2/25/2020	Saura C (2020)^134^
						Arm B: lapatinib (1250 mg qd) + capecitabine (2000 mg/m^2^ daily, 1000 mg/m^2^ bid)	26.7%	5.5 mo	18.7 mo		
	Tucatinib	HER2CLIMB trial (NCT02614794)	II	612	HER2-positive metastatic breast cancer who had prior treatment with trastuzumab, pertuzumab, and ado-trastuzumab emtansine	Tucatinib 300 mg bid plus trastuzumab and capecitabine (N=410)	40.6%	7.8 mo	21.9 mo	4/17/2020	Murthy R, (2018, 2020)^60^^,^^61^
						Placebo plus trastuzumab and capecitabine (N=202)	22.8%	5.6 mo	17.4 mo		
**Monoclonal Antibody**	Trastuzumab	NA	III	469	Progressive metastatic breast cancer that overexpressed HER2 who had not previously received chemotherapy	Chemotherapy plus trastuzumab	50%	7.4mo	25.1 mo	9/25/1998	Slamon DJ (2001)^135^
						Chemotherapy	32%	4.6 mo	20.3 mo		
		HERA trial (NCT00045032)	III	5099	HER2-overexpressing primary breast cancer who have completed (neo-)adjuvant systemic chemotherapy, definitive surgery, and radiotherapy	1 or 2-year trastuzumab	Estimates of 10-year disease-free survival: 69%				Cameron D (2017)^136^
						Observation or placebo?	Estimates of 10-year disease-free survival: 63%				
	Pertuzumab	CLEOPATRA (NCT00567190)	III	808	HER2-positive metastatic breast cancer	Pertuzumab plus trastuzumab and docetaxel (N=402)	80.2%	18.7 mo; HR 0.69 (95% CI: 0.58, 0.81)	57.1 mo; HR 0.69 (95% CI: 0.58, 0.82)	6/8/2012	Baselga J (2012) Swain SM (2013, 2020^137^, ^138^, ^139^
						Placebo plus trastuzumab and docetaxel (N=406)	69.3%	12.4 mo	40.8 mo (95% CI: 36, 48)		
**Drug conjugate**	Ado-trastuzumab emtansine	EMILIA (NCT00829166)	III	991	HER2-positive locally advanced or metastatic breast cancer	Ado-trastuzumab emtansine	43.6%	9.6 mo	30.9 mo	2/22/2013	Verma S (2012)^140^
						Lapatinib plus capecitabine	30.8%	6.4 mo	25.1 mo		
		KATHERINE (NCT01772472)	III	1486	HER2-positive early breast cancer	Ado-trastuzumab emtansine 3.6 mg/kg IV	88.3%	3-year-IDFS		5/6/2019	von Minckwitz G (2019) Modi S^141^
						Trastuzumab 6 mg/kg IV q3w for 14 cycles	77%				
	Trastuzumab deruxtecan (DS-8201)	DESTINY-Breast01 (NCT03248492)	II	184	Advanced HER2-positive breast cancer	Trastuzumab deruxtecan (5.4 mg/kg) IV three different doses	60.9%	16.4 mo;19.4 mo (95% CI: 14.1, NE)	24.6 mo (95% CI: 23.1, NE)	12/9/2019	Modi S (2020)^74^^,^^142^ Mauro G (2020)^143^
	Margetuximab-cmkb	SOPHIA Study (NCT02492711)	III	536	Advanced pretreated HER2-positive (IHC 3^+^ or FISH) metastatic breast cancer	Margetuximab plus chemotherapy, q3w (N=268)	22% (95% CI: 17, 27)	5.8 mo (95% CI: 5.5, 7.0)	Median DOR 6.1 mo (95% CI: 4.1, 9.1)	12/16/2020	Rugo HS (2019)^97^^,^^98^
											
						Trastuzumab plus chemotherapy, q3w (N=268)	16% (95% CI: 12, 20)	4.9 mo (95% CI: 4.2, 5.6)	Median DOR 6.0 mo (95%CI: 4.0, 6.9)		

Abbreviations: bid, twice daily; CI, confidence interval; DFS, disease-free survival; DOR, duration of response; HR, hazard ratio; IDFS, invasive disease-free survival; IV, intravenously; mo, month; NA, not available; NE, not estimable; NR, not reached; ORR, overall response rate; OS, overall survival; PFS, progression free survival; qd, once daily; q3w, every 3 weeks; wk, week.

### 4.1 Small molecule tyrosine kinase inhibitors (TKIs)

Small molecule TKIs enter the cell and bind to the cytoplastic ATP binding site at the tyrosine kinase domain to prevent phosphorylation and activation of the receptor. Monoclonal antibodies, in contrast, function extracellularly by inhibiting ligand binding and/or homo/heterodimerization of the receptor with activation of the signaling pathway (Fig. 2). To date, no TKI that exclusively targets HER2 has been developed. On the other hand, selective EGFR TKIs such as osimertinib were successfully developed against EGFR. There are two classes of EGFR/HER2 TKIs: those that bind reversibly (such as lapatinib) and those that bind irreversibly (covalently) (such as afatinib, decomtinib) to the tyrosine kinase domain ATP binding site. Among these available drugs, afatinib is the most commonly used off-label TKI for *HER2*-amplified or mutated NSCLC^35^^,^^36^. In the most comprehensive preclinical study to date, common *HER2* mutants in exons 19–21 show various sensitivities to eleven EGFR/HER2 TKIs *in vitro*. Poziotinib was the most potent *HER2*-mutant selected TKI tested, both alone or in combination with ado-trastuzumab emtansine^15^. However, the clinical development of poziotinib has been hampered by modest clinical activity with significant gastrointestinal and dermal toxicities as monotherapy^37^. Recently, a newer generation of pan-HER TKIs, such as pyrotinib, mobocertinib, tucatinib, BDTX-189, and tarloxotinib, show promising preclinical and clinical activity that is reviewed below.

#### 4.1.1 Afatinib

Afatinib (BIBW2992) is a highly selective, potent, irreversible inhibitor for EGFR, HER2 and HER4 kinases^38^^,^^39^. The irreversible tyrosine kinase blockade may result in longer suppression of HER2 signaling compared to reversible inhibitors^40^^,^^41^. In a single arm, phase II study (NICHE), afatinib achieved disease control at 12 weeks in 53.8% of 13 patients with advanced NSCLC harboring *HER2* exon 20 mutations. Median progression free survival (PFS) and overall survival (OS) were 15.9 weeks (95% CI: 6.0, 35.4) and 56 weeks (95% CI: 16.3, not reached), respectively^42^. In a retrospective study, 23 patients with stage IV or recurrent HER2 mutated lung adenocarcinomas were treated with afatinib^43^. Of the 23 patients, 13% of patients had a partial response (PR), 57% had stable disease (SD), and 30% showed disease progression. The median response time to afatinib was 6 months and median OS was 23 months. A preclinical study showed that afatinib in combination with mammalian TOR (mTOR) inhibitor rapamycin was the most effective way to inhibit the tumor growth in HER2 YVMA-mutant transgenic NSCLC mice when compared to trastuzumab and/or rapamycin treatment. Both upstream and downstream MAPK and Akt/mTOR axes were inhibited by afatinib and rapamycin combination^40^. A phase Ib study showed that afatinib combined with either intravenous vinorelbine or oral vinorelbine was safe in EGFR/HER2 overexpressed solid tumors^44^. Among the evaluated patients, 16 out of 28 (57.1%) patients in the intravenous group showed SD, and 3 out of 27 (11.1%) patients in the oral group achieved PR. The median PFS was 14.6 and 15.9 weeks in intravenous and oral group, respectively. Afatinib has different sensitivities in different HER2 mutation variants in NSCLC. In a large retrospective analysis containing 118 patients with HER2-mutated metastatic NSCLC, a total of 31 HER2 mutation variants and 35 concomitant genomic alterations were detected. This suggests HER2-mutant NSCLC is a group of very heterogenous disease. Certain variants, G778_P780dup and G776delinsVC, derive sustained clinical benefits from afatinib. Whereas the predominant variant, A775_G776insYVMA, is resistant to most available anti-HER2 treatments including afatinib^28^. Because it is commercially available for patients with EGFR-mutant NSCLC, afatinib is used in clinical settings with various responses. In some cases, this includes PR or SD for patients with HER2 mutations (including A775_G776insYVMA)^28^^,^^45^^,^^46^.

#### 4.1.2 Dacomitinib

Dacomitinib (PF-00299804) is an irreversible pan-HER TKI that inhibits the kinase activity of wild-type EGFR, HER2, and HER4*.* Furthermore, it demonstrates an effect against gefitinib resistant NSCLC models harboring either *HER2* amplifications or mutations in preclinical studies^47^. Dacomitinib is approved as an option for first line systemic therapy in patients with *EGFR*-mutant, advanced NSCLC cancer^48^. The median PFS was 14.7 months in the oral dacomitinib 45 mg/day group compared to 9.2 months in the oral gefitinib 250 mg/day group. The median OS was 34.1 months in dacomitinib group versus 26.8 months in gefitinib^49^. A phase II study of 26 patients with HER2-positive, advanced NSCLC, dacomitinib led to PR in 3 of 26 (11.5%) patients with *HER2* mutations (25 insertion and 1 missense mutation) compared to no PR observed in 4 patients with *HER2* amplification. The median OS was 9 months in the *HER2* mutation group. OS ranged from 5 to 22 months in the *HER2* amplification group^17^. Similar to afatinib, dacomitinib is used by clinicians for HER2-positive NSCLC patients when clinical trials are not available, or patients are not candidates or decline systemic chemotherapy.

#### 4.1.3 Pyrotinib

Pyrotinib is an oral, irreversible pan-HER TKI with strong activity against EGFR and HER2^50^. Pyrotinib first received approval in China for metastatic breast cancer treatment^51^. Despite similar preclinical activity, pyrotinib has superior clinical activity compared to lapatinib in combination with capecitabine for patients with metastatic breast cancer (mBCA)^52^. Preclinical and phase 1 studies suggest that pyrotinib could effectively inhibit the proliferation of HER2-overexpressing cells *in vitro* and *in vivo*^53^^,^^54^. In *HER2* exon 20 mutant NSCLC patient-derived organoid or xenograft models pyrotinib has shown potent tumor growth inhibition. This is achieved through inhibition of phosphorylated HER2 and downstream phosphorylated ERK and AKT pathways^50^. During early phase clinical trials, pyrotinib demonstrated promising clinical activity with good tolerability in both Chinese and US patients. In the Chinese cohort, among 28 enrolled HER2-positive metastatic breast cancer patients, 60.7% were trastuzumab-pretreated patients. The overall response rate (ORR) was 78.6% and median PFS of 22.1 months. The ORR was 90.9% and 70.6% in trastuzumab-naïve and trastuzumab-pretreated patients, respectively^55^. In the U.S. cohort, mean prior treatment was 3. Although 26% of the patients received more than 3 lines of prior therapy including HER2 targeted treatments (e.g. trastuzumab, ado-trastuzumab emtansine and other HER2 TKIs), the median PFS was 5.4 months and ORR was 13%^56^. Of 4 patients with confirmed PR and 5 patients with SD for more than 6 months, all had *HER2* exon 20 mutations. Further analysis showed 6 cases with A775_G776insYVMA, 1 with E770A-A771insAYVM, 1 with G778-P780dup, and 1 with G778_779insCPG. The most common treatment emergent adverse effect was diarrhea with grade 3 and 4 toxicity present in 24.3% of patients. A phase II clinical trial (NCT02834936) enrolled 60 pretreated *HER2-*mutant NSCLC patients. Subjects received 400 mg of pyrotinib daily. The observed ORR was 31.7% (95% CI: 20.3%, 45.0%), median duration of response (DOR) and PFS were 7.0 months (95% CI: 5.5, 11.0) and 6.8 months (95% CI: 4.1, 8.3), respectively^57^. Prophylactic antidiarrheal treatment with loperamide was recommended. Based on these results, a phase III, global, randomized clinical trial (NCT04447118) comparing the efficacy of pyrotinib to docetaxel as second line systemic therapy in patients with advanced, non-squamous NSCLC harboring *HER2* exon 20 mutations) who have failed platinum-based chemotherapy was recently opened to enroll patients in September 2020.

#### 4.1.4 Mobocertinib

Mobocertinib (TAK-788) is an oral TKI with potent preclinical activity selectively against *EGFR* and *HER2* mutations, including exon 20 insertions. A phase I/II clinical trial enrolled 34 patients with previously treated, advanced NSCLC with *EGFR/HER2* exon 20 insertions or mutations. Patients received oral mobocertinib in daily doses of up to 180 mg during the dose-escalation phase^58^. Among patients with *EGFR* exon 20 insertions who received 80- to 160-mg doses of mobocertinib daily, 39% responded and 94% had radiographically demonstrated disease control. Adverse effects were mostly mild and frequently included diarrhea, nausea, fatigue, and rash. These symptoms were similar to those with other TKIs. Investigators plan to study a 160-mg daily dose of mobocertinib in a phase 2 expansion cohort. Based on these results, the U.S. FDA granted mobocertinib Orphan Drug Designation for the treatment of lung cancer with *HER2* mutations or *EGFR* mutations (including exon 20 insertion) in 2019. Additionally, mobocertinib received a breakthrough therapy designation for *EGFR* exon 20 insertion mutation NSCLC in 2020^59^. The efficacy of mobocertinib for patients with locally advanced or metastatic NSCLC whose tumors harbor *HER2* exon 20 insertions has not been reported.

#### 4.1.5 Tucatinib

Tucatinib is the newest oral HER2 TKI approved for metastatic HER2-positive breast cancer. *In vitro* tucatinib inhibits phosphorylation of HER2 and HER3, which results in blockade of downstream PI3K/AKT and Ras/MAPK signaling pathways, thus inhibiting cell growth^60^^,^^61^. Although *in vivo*, tucatinib monotherapy demonstrates limited preclinical activity^62^. Tucatinib in combination with trastuzumab and capecitabine was approved by the US FDA for adult patients with advanced unresectable or metastatic HER2-positive breast cancer in April 2020. This includes patients with brain metastases who have received one or more prior anti-HER2-based regimens in the metastatic setting. In 6 *HER2*-mutant patient derived xenograft models for colorectal, NSCLC, gallbladder and gastric cancers tucatinib and trastuzumab demonstrated promising synergistic antitumor effects in those harboring L755S, V77L or S310Y mutations^63^. Tucatinib and trastuzumab inhibited the growth of HER2 L755S mutations in NSCLC. In human lung cancer cell line NCI-H1781 it was observed that tucatinib can inhibit HER2 phosphorylation. A basket trial of tucatinib and trastuzumab (NCT04579380) is evaluating the clinical activity in solid tumors with HER2 alterations, including a cohort for NSCLC (accrual start date: January 11, 2021).

#### 4.1.6 BDTX-189

A new *EGFR/HER2* mutation selective TKI, BDTX-189, received the FDA Fast Track Designation in July 2020. BDTX-189 is an orally available, irreversible small molecule inhibitor that is designed to spare wildtype EGFR while blocking the function of EGFR and HER2 kinase domain exon 20 insertions. Additionally, it is designed to blockade other activating oncogenic drivers of HER2, including the S310F/Y mutation. Similar to the third generation EGFR TKI osimertinib, the sparing of wild-type EGFR by BDTX-189 has the potential to improve the toxicity profile of current HER2 kinase inhibitors and has high permeability into the brain^64^. The MasterKey-01 study (NCT04209465), a phase I/II trial of BDTX-189 is recruiting adult patients with solid tumors harboring an allosteric *HER2* mutation, or an *EGFR* or *HER2* Exon 20 insertion mutation, who have progressed following prior treatment and lack alternative therapy options.

#### 4.1.7 Tarloxotinib

Tarloxotinib is the hypoxia-activated prodrug of a pan-ErbB kinase inhibitor that releases a potent irreversible active metabolite (tarloxotinib-E). This metabolite is preferentially delivered to the active moiety of tumor versus normal tissues. Tarloxotinib demonstrated preclinical efficacy in *EGFR* exon 20, *HER2*-mutant NSCLC, *HER2*-amplified NSCLC, and other oncogenic alterations in the ERBB gene family (such as NRG1 fusions)^65^. In July 2020, a phase II clinical trial (RAIN-701 trial, NCT03805841) evaluated 11 *HER2*-mutant NSCLC patients who failed platinum-based chemotherapy to be treated with tarloxotinib. Four out of 9 evaluable patients demonstrated tumor shrink, 22% patients had PR, and 44% patients had SD^66^. Three patients remained on treatment after 6 months. Thus, tarloxotinib shows promising clinical efficacy in patients with *HER2*-mutant NSCLC.

### 4.2 The role of chemotherapy in HER2-positive NSCLC

HER2-targeted therapies are well tolerated but lack clinical efficacy in HER2-positive NSCLC. Therefore, several clinical trials evaluated the efficacy of various chemotherapy in combination with HER2 targeting agents. A phase II trial evaluated the efficacy of carboplatin, paclitaxel and trastuzumab in patients with HER2-positive NSCLC. Of 139 patients screened, 53 were enrolled with HER2 positivity graded by IHC staining from 1^+^ to 3^+^. The combination of chemotherapy and trastuzumab was well tolerated. For IHC 1^+^ and 2^+^ patients, the OS was observed to be consistent with historical data using carboplatin and paclitaxel alone. Patients with IHC 3^+^ HER2 had higher OS compared to IHC 1^+^ and 2^+^ patients, suggesting a potential benefit to trastuzumab therapy in this subset^67^. Similarly, a randomized phase II trial assessed the addition of trastuzumab to cisplatin/gemcitabine in previously untreated patients with HER2-positive NSCLC. Of the 619 patients screened, 103 patients were tested positive for HER2 and received gemcitabine and cisplatin with trastuzumab versus gemcitabine and cisplatin alone. Again, no clear clinical benefit was observed in the trastuzumab treated group versus chemotherapy alone. Although some benefit was observed in the HER2 IHC 3^+^ group, the subset was too small to provide definitive information^68^. Lastly, the addition of trastuzumab to weekly docetaxel had a PR rate of 8% in a small phase II trial with 13 patients who had IHC 2^+^ or 3^+^ HER2 positive tumors and progression after platinum-based chemotherapy^69^. Overall, the results support further development of HER2-targeted therapy and chemotherapy.

### 4.3 HER2-targeted antibody drug conjugates

#### 4.3.1 Ado-tratuzumab emtansine (T-DM1)

Ado-tratuzumab emtansine (T-DM1) is a HER2 targeting antibody-toxin conjugate which is composed of trastuzumab, a stable thioether linker, and the potent cytotoxic agent DM1^70^. Preclinical studies demonstrated that ado-trastuzumab emtansine combines the distinct mechanisms of action of both trastuzumab and DM1. Furthermore, clinical experiments show a minimal amount of systemic exposure to free DM1, without evidence of DM1 accumulation after repeat doses. In a phase II basket trial^71^, 18 advanced *HER2*-mutant lung adenocarcinomas received ado-trastuzumab emtansine and had a PR rate of 44% with median PFS of 5 months. Another phase II trial evaluated ado-trastuzumab emtansine efficacy in previously treated HER2- overexpressing NSCLC^72^. Forty-nine patients were divided into HER2 immunohistochemistry (IHC) 2^+^ and IHC 3^+^ groups. None in the IHC 2^+^ group showed response to ado-trastuzumab emtansine, and 4 out of 20 patients with IHC 3^+^ expression showed PR. The ORR was 20%. Clinical benefit rates were significantly higher in the IHC 3^+^ group, 30% vs 7%. The response duration in those four responders range from 2.9 to 10.8 months. Of note, 3 out of 4 responders also had *HER2* amplification. Currently, ado-trastuzumab emtansine is recommended by NCCN guidelines for advanced NSCLC with *HER2* mutation (category 2A)^3^.

#### 4.3.2 Trastuzumab deruxtecan

Trastuzumab deruxtecan (also known as fam-trastuzumab deruxtecan-nxki, fam-trastuzumab deruxtecan, T-DXd, DS-8201a, or DS-8201) is an antibody-drug conjugate composed of a direct anti-HER2 antibody, trastuzumab, and topoisomerase inhibitor^73^. Trastuzumab deruxtecan has a higher payload with an 8 to 4 chemotherapy drug-to-antibody ratio than tratuzumab emtansine with a 3 to 4 drug-to-antibody ratio. Based on the results of DESTINY-Breast01 (NCT03248492)^74^, trastuzumab deruxtecan was approved by the U.S. FDA for patients with late stage HER2-positive breast cancer as second line treatment on December 20, 2019. A phase II clinical trial (DESTINY-Gastric01, NCT03329690) included 188 HER2-positive late stage gastric or GEJ adenocarcinoma patients. Subjects were randomly (2:1) assigned to receive trastuzumab deruxtecan 6.4 mg/kg/q3w intravenously or either irinotecan vs paclitaxel monotherapy^75^. OS was observed to be 12.5 months (95% CI: 9.6, 14.3) and 8.4 months (95% CI: 6.9, 10.7) in the trastuzumab deruxtecan arm and the irinotecan or paclitaxel arm (HR=0.59; 95% CI: 0.39, 0.88, *P*=0.0097) respectively. 40.5% (95% CI: 31.8, 49.6) confirmed ORR was observed in the trastuzumab deruxtecan arm compared with 11.3% (95% CI: 4.7, 21.9) in the irinotecan or paclitaxel arm. Median PFS was 5.6 months (95% CI: 4.3, 6.9) and 3.5 months (95% CI: 2.0, 4.3) in trastuzumab deruxtecan arm and the irinotecan or paclitaxel arm respectively. Median DOR was 11.3 months (95% CI: 5.6, NR) in the trastuzumab deruxtecan arm and 3.9 months (95% CI: 3.0, 4.9) in the irinotecan or paclitaxel arm. Based on these results, the U.S. FDA approved trastuzumab deruxtecan for patients with locally advanced or metastatic HER2-positive gastric or GEJ adenocarcinoma who failed prior trastuzumab-based treatment on January 15, 2021^76^.

Trastuzumab deruxtecan also demonstrated promising clinical activity in patients with *HER2*-positive metastatic NSCLC. In an ongoing phase 1 trial, trastuzumab deruxtecan (6.4 mg/kg) had a confirmed ORR of 58.8% (10/17) in *HER2*-expressing or mutated NSCLC, and 72.7% (8/11) in *HER2*-mutated NSCLC, with a manageable safety profile^77^. The first interim analysis from the ongoing phase II DESTINY-Lung01 trial (NCT03505710) was first presented at the ASCO 2020 Virtual Scientific Program^78^. The results showed that trastuzumab deruxtecan had a confirmed ORR of 61.9% (95% CI: 45.6%, 76.4%) in 42 patients with *HER2*-mutated NSCLC. This included 1 patient (2.4%) with a complete remission and 59.5% of patients with a PR. Additionally, 28.6% of patients had SD. Thus, the disease control rate was 90.5% (95% CI: 77.4%, 97.3%). For most patients (90.5%), their *HER2* mutations were located in the kinase domain. Only 1 patient had a mutation present in the extracellular domain, and 1 patient did not have this information reported. All patients reported treatment-emergent adverse events (TEAEs), and among them 64.3% were over grade 3. The overall safety and tolerability profile of trastuzumab deruxtecan was consistent with observations seen in the phase I trial. The most common adverse events to date (n=42) were gastrointestinal and hematological, including nausea, alopecia, anemia, decreased appetite, and decreased neutrophil count. Drug-related interstitial lung disease was observed in 5 cases, and all of them were grade 2^78^. Based on these results, trastuzumab deruxtecan received the breakthrough therapy designation for *HER2*-mutant metastatic NSCLC from the U.S. FDA in May 2020^79^. It was recently added to ado-trastuzumab emtansine for *HER2* mutated NSCLC in the NCCN guidelines V1.2021. The activity of T-DXd patients with HER2 overexpression cohort was recently reported in WCLC 2020^80^. The ORR was 24.5%, the median PFS and median OS in HER2 overexpression by IHC cohort was reported as 5.4 months (95% CI: 2.8, 7.0 months) and 11.3 months (95% CI: 7.8, not evaluable) respectively. Compared to the HER2-mutant cohort, trastuzumab deruxtecan had lower ORR, PFS and OS in the HER2 overexpressed cohort by immunohistochemistry (IHC)^80^. Similar data were observed for afatinib^36^ and ado-trastuzumab emtansine^71^^,^^72^, suggesting *HER2*-mutant and *HER2*-amplified NSCLC are distinct entities. Together with the variations in concurrent genomic alterations, further biomarker study is needed to select the appropriate patients with HER2-positive NSCLC.

Recently, HER2-targeted therapy has been expanded to include chimeric antigen receptor (CAR) T-cell therapy in lung cancer. Additionally, several new HER2-targeting drugs are in early phases of clinical development. Table 5 summarizes several ongoing studies for targeting HER2 alterations in NSCLC. Among these studies, ongoing phase II DESTINY-Lung01 trial (NCT03505710) and the newly activated phase III study of pyrotinib after first line platinum-based chemotherapy are the most advanced.

**Table 5:** Ongoing clinical trials of HER2-targeted agents for patients with metastatic NSCLC.

**ClinicalTrials.Gov Identifier**	**Biomarker: HER2 mutation, amplification, or high IHC expression**	**Regimen**	**Phase**	**Sample Size**	**Estimated or Actual Study Period**		
NCT03505710(DESTINY-Lung01)	HER2 overexpression or *HER2* mutation	Trastuzumab deruxtecan (DS-8201) 6.4 mg/kg or 5.4 mg/kg	II	170	May 21, 2018-August 31, 2021		
NCT04447118(PYRAMID-1)	*HER2* exon 20 mutations	Pyrotinib 400 mg qd ordocetaxel 75 mg/m q3w	III	150	September 30, 2020- October 31, 2023		
NCT02465060	1. *HER2* activating mutations2. *HER2* amplification ≥7 copies	AfatinibPertuzumabTrastuzumab	II	6452	August 12, 2015-June 30, 2022		
NCT02716116	*HER2* exon 20 activating insertions or point mutations and active or *EGFR* exon 20 mutation	Mobocertinib (TAK-788)	I/II	306	June 16, 2016- March 13, 2023		
NCT02183883	*EGFR* and *HER2* mutations	Afatinib 40 mg, 30 mg, 20 mg, qd	II	48	December 16, 2016- November 2023		
NCT03318939	*EGFR* or *HER2* exon 20 insertion mutations	Poziotinib	II	603	October 13, 2017- December 30, 2023		
NCT03574402 (Guangdong, China)	*HER2* mutations, Arm 7 in the Umbrella Study Directed by Next Generation Sequencing (TRUMP)	Pyrotinib 400 mg qd	II	400 (one arm)	July 9, 2018-December 30, 2024		
NCT04063462 (Tianjin, China)	*EGFR* or *HER2* exon 20 insertion mutations	Pyrotinib, 400 mg qd	II	60	October 1, 2019-October 1, 2021		
NCT03805841	*HER2*-activating or *EGFR* exon 20 insertion mutations	Tarloxotinib bromide	II	60	March 13, 2019- March 15, 2021		
NCT03974022	*EGFR* or *HER2* mutations	DZD9008 50 mg qd	I/II	160	July 9, 2019-March 2023		
NCT04209465 (MasterKey-01)	Phase I: Solid tumor patients with: 1. Allosteric *HER2* or *HER3* mutation(s); 2. *EGFR* or *HER2* exon 20 insertion mutation(s); 3. *HER2* amplified or overexpressing tumors; 4. *EGFR* exon 19 deletion or L858R mutation.Phase II: Solid tumor patients with: 1. Allosteric *HER2* mutation; 2. *EGFR* or *HER2* exon 20 insertion mutation.	BDTX-189	I/II	200	December 19, 2019-December 2023		
NCT04402008	*EGFR* or *HER2* exon 20 insertion mutations	Poziotinib qd or bid	II	40	June 26, 2020- March 2025		
NCT02892123	HER2 1^+^, 2^+^ or 3^+^ by IHC	ZW25 (Zanidatamab)Paclitaxel, Capecitabine, Vinorelbine	I	234	September 2016-March 31, 2022		
NCT04460456	HER2 IHC 2^+^ or 3^+^	SBT6050	I	210	July 27, 2020- August 2024		
NCT04464967	HER2 or EGFR-positive by IHC	Ex Vivo Expanded, Autologous Natural Killer Cells (SNK01) in combination of trastuzumab and cetuximab	1/2a	154	August 2020- February 2023		
NCT02675829	*HER2* amplification or mutation	Ado-trastuzumab emtansine	II	100	February 2016- February 2021		
NCT02314481	*HER2* amplification	Trastuzumab emtansine 3.6 mg/kg q3w	II	119	May 12, 2017- November 2025		
NCT03602079	HER2 positive (by ISH or NGS) or *HER2* amplification	A166	I/II	82	July 16, 2018- May 2021		
NCT04311034	HER2 overexpression (IHC 2^+^/3^+^) or mutation	RC48-ADC 2.0 mg/kg/q2w	Ib	36	September 26, 2018-December 30, 2021		
NCT03821233	HER2-expression by IHC	ZW49	I	174	April 15, 2019-April 30, 2023		
NCT03198052	HER2, Mesothelin, Lewis-Y, PSCA, MUC1, GPC3, AXL, EGFR, or B7-H3	CAR-T cells targeting HER2, Mesothelin, PSCA, MUC1, Lewis-Y, GPC3, AXL, EGFR or B7-H3	I	30	July 1, 2017- August 1, 2023		
NCT03500991	HER2 ^+^ by IHC	HER2-specific CAR-T cell	I	48	July 26, 2018- July 26, 2036		
NCT03696030	HER2 ^+^ by IHC or *HER2* gene amplification by FISH	HER2 CAR-T cells	I	39	August 31, 2018- August 31, 2021		
NCT04319757	HER2 expression by IHC	ACE1702	I	24	May 19, 2020-June 2022		
NCT04511871	HER2 IHC 3^+^	CCT303-406 cells	I	15	July 9, 2020-January 31, 2023		
NCT03740256	HER2 positive by IHC	HER2 specific CAR-T cells	I	39	September 1, 2020- September 1, 2038		
NCT00906698	Overexpress HER2 and/or EGFR	Afatinib 20, 40 or 50 mg qd + IV vinorelbine 25 mg m^−2^ q1w or oral vinorelbine 60/80 mg m^−2^ q1w	I	55	April 17, 2014- June 9, 2014		
NCT03810872	*EGFR, HER 2* and *HER3* mutations	Afatinib 40 mg/qd + paclitaxel 80 mg/kg/3w	II	87	June 21, 2017- December 2022		
NCT03784599	HER2-overexpression (IHC≥2^+^/ FISH) or activating *EGFR* mutation	Trastuzumab emtansine 3.0 mg/kg IV q3w + osimertinib qd	II	58	December 18, 2018- April 1, 2022		
NCT04144569	*HER2* insertion mutations	PD-1 combined with pyrotinib	II	30	January 30, 2019- December 31, 2020		
NCT03845270	*HER2* exon 20 mutations or insertions	pertuzumab + trastuzumab + docetaxel 75 mg/m²	II	45	May 17, 2019- March 2022		
NCT04042701	HER2-overexpression (IHC or FISH) or *HER2*-mutations	Trastuzumab deruxtecan (DS-8201a) + pembrolizumab	Ib	115	February 10, 2020-April 2022		
NCT04382300	*HER2* mutations	Pyrotinib 400 mg qd, combined with thalidomide 200 mg qd	II	39	May 2020- April 2023		

Abbreviations: bid, twice daily; CAR-T cell, chimeric antigen receptor T cell; CNS, central nervous system; FISH, fluorescence in situ hybridization; IHC, immunohistochemistry; IV, intravenously; NGS, next-generation sequencing; od, every other day; qd, once daily; q2w, every 2 weeks; q3w, every 3 weeks. Blue highlights two most advanced studies for HER2-positive NSCLC.

### 4.4 Systemic therapy with immune checkpoint inhibitor therapy

In a retrospective study of 122 patients with *HER2*-mutant lung cancer, programmed cell death-ligand 1 (PD-L1) expression was lower but tumor mutation burden (TMB) was similar to those in unselected lung cancers^81^. There are only a few retrospective studies containing *HER2*-mutant NSCLC patients treated with immune checkpoint inhibitors, each with about 20–30 cases^81^, ^82^, ^83^, ^84^, ^85^. Patients with *HER2*-mutant NSCLC had an ORR of 6%-7.4% and a median PFS of 1.9–2.5 months with second-line immune checkpoint inhibitors^82^^,^^83^. This is inferior to an ORR of 10% and a median PFS of 4.3 months with second-line non-HER2 targeted chemotherapies^84^. None of the responders had a *HER2* exon 20 YVMA mutation. In another retrospective study involving 23 patients with *HER2*-mutant NSCLC, responses were seen in six patients (27.3%), but the effect is short lived with a median PFS of 2.2 months and an OS of 20.4 months^85^. Of note, in the unselected platinum-refractory NSCLC population, standard second-line chemotherapy, docetaxel, has an ORR of 7–13% and a median PFS of 2–4 months^3^. Therefore, platinum-based chemotherapy with or without an anti-angiogenesis inhibitor remains the first line standard treatment for this patient population. Further study is needed to define the role of immune checkpoint inhibitors in *HER2*-mutant NSCLC.

## 5 Resistance mechanisms of HER2-targeted therapy

Unfortunately, many patients that received HER2-targeted therapy in NSCLC develop drug resistance in less than 6 months. There are unmet needs to both understand resistance mechanisms and to develop subsequent treatment strategies to overcome the resistance to HER2-targeted therapy. Until now, most of the data have been from studies in breast cancer. Both HER2-dependent and HER2-independent resistance mechanisms were reported in NSCLC patients who developed resistance to HER2-targeted therapies. One of the independent resistance mechanisms is activation of compensatory pathways including reactivation of the PI3K/AKT and Ras/MAPK signaling pathways^24^. It was reported that PTEN-deficiency contributes to trastuzumab resistance in breast cancer patients and inhibition of PI3K could rescue PTEN-deficiency induced resistance^86^. Src activation, L755S mutation and T798I mutation were also reported in mediating drug resistance to HER2-targeted drugs including trastuzumab, lapatinib and neratinib^87^, ^88^, ^89^. Co-genomic alterations also contribute to HER2 drug resistance. For example, cyclin E overexpression was associated with trastuzumab resistance in breast cancer patients^90^, and a high level of *HER2* somatic copy-number alterations (SCNAs) was correlated with innate trastuzumab resistance during disease progression^91^. In addition, HER2 mutations, such as L755S, V777L, D769Y, V842I, K753E, I655V, have been reported in resistance cases^92^. HER2 kinase domain mutation resulting in constitutive activation of HER2 and EGFR were described in HER2/EGFR drug resistance cases^93^.

Concurrent *HER2* amplification has been reported in 3–5% of patients with *EGFR*-mutant NSCLC, which was an independent predictor of shorter time to progression (HR=2.4, *P*=0.015) on EGFR TKI treatment. This can be attributed the activation of HER2 share the same downstream network with EGFR, which is associated with very aggressive biology and poor clinical outcomes in NSCLC^94^. *HER2* genomic alterations are identified as a primary or acquired resistance mechanism to first generation EGFR TKI (erlotinib) and second generation EGFR TKI (afatinib) in NSCLC^95^. Recently, the *HER2* signaling pathway is also recognized as a driver of resistance to the third generation TKI osimertinib. For instance, the exon 16-skipping splice variant of *HER2* (HER2D16) is a mediator of osimertinib resistance in EGFR L858R/T790M lung cancer. Combining afatinib and osimertinib could overcome HER2D16-mediated resistance^96^. Ongoing clinical trials (NCT04464967, NCT04144569) are evaluating the addition of a HER2 inhibitor to an EGFR inhibitor, or an immune checkpoint inhibitor, to overcome the acquired resistance in patients with *HER2*-mutated NSCLC.

## 6 Summary and perspective

Clinical application of multiplex biomarker testing by NGS identifies rare oncogenic mutations and amplification in the *HER2* gene as two major genomic alterations in NSCLC. The most common *HER2* mutation is in exon 20 A775_G776insYVMA. In patients with previously untreated or treated NSCLC, this mutation is associated with sensitivity to several HER2-targeting drugs including afatinib, dacomitinib, pyrotinib, BDTX-189, tarloxotinib, ado-trastuzumab emtansine, and trastuzumab deruxtecan. Unlike in breast and gastrointestinal cancer, currently available HER2-targeted agents only have modest clinical activity in *HER2*-mutant or -amplified NSCLC. Trastuzumab deruxtecan received the U.S. FDA breakthrough therapy designation for *HER2*-mutant metastatic NSCLC in May 2020. Two randomized studies are in late-stage clinical development. A global phase III study of pyrotinib compared to docetaxel as a second line therapy for advanced NSCLC harboring *HER2* exon 20 mutations (PYRAMID-1, NCT04447118) was just opened for enrollment in September 2020. A randomized phase II study of tarloxotinib versus platinum-based chemotherapy as a first line systemic therapy is also actively recruiting patients with advanced NSCLC harboring *HER2* exon 20 mutations (RAIN-701 trial, NCT03805841). Ongoing studies are also evaluating the feasibility and clinical benefit of HER2-targeted therapy in combination with other agents in advanced NSCLC patients. There are unmet needs to understand the biology of *HER2* genomic alterations, to optimize biomarker testing, and to determine the resistance mechanisms of both primary and acquired resistance for patients with *HER2*-altered NSCLC.

## Declaration of Competing Interest

Dr. Li reports personal fees from Eisai, grants from Pfizer, grants from Merck, grants from Eureka, grants from OncoImmune, grants from Hengrui, grants from Tempus, outside the submitted work.
